# Cardiopulmonary Failure Requiring ECMO Bypass Resulting from Leukemia Cell Lysis in a Patient with Childhood Acute Myelomonocytic Leukemia

**DOI:** 10.1155/2015/640528

**Published:** 2015-06-01

**Authors:** Michael Huang, Erin Owen, Scott Myers, Ashok Raj

**Affiliations:** ^1^Division of Pediatric Hematology, Oncology and Stem Cell Transplantation, University of Louisville, Louisville, KY, USA; ^2^Division of Pediatric Critical Care Medicine, University of Louisville, Louisville, KY, USA

## Abstract

*Background*. Childhood AML patients are at increased risk for early fatal pulmonary complications. Pulmonary leukostasis and systemic inflammatory response syndrome (SIRS) following leukemia cell lysis are the likely etiologies. *Observation*. Soon after initiation of AML chemotherapy, an 18-month-old female who met SIRS criteria sustained cardiopulmonary failure requiring ECMO support. Upon recovery, the patient went on to complete therapy and remains in remission without permanent neurologic or cardiac sequelae. *Conclusion*. Cytokine release syndrome from rapid cell lysis was the likely cause as infectious workup failed to reveal a definitive etiology and drug hypersensitivity testing to the chemotherapy agents was negative.

## 1. Background

Patients with childhood acute myelogenous leukemia (AML) are at increased risk for early death secondary to pulmonary complications. Pulmonary leukostasis and systemic inflammatory response syndrome (SIRS) following leukemia cell lysis have been implicated as the likely etiologies. We herein report the case of an 18-month-old female who sustained cardiopulmonary failure requiring Extracorporeal Membrane Oxygenation (ECMO) support following initiation of chemotherapy for acute myelomonocytic (M4eo) leukemia.

## 2. Case Report

The patient was an 18-month-old female with AML M4eo. She presented with fever and left calf cellulitis and was placed on IV clindamycin. Her CBC at the time showed circulating blasts with WBC 17.51 × 10^9^/L, Hgb 9.2 g/dL, and platelets 88 × 10^9^/L. Bone marrow aspiration showed 15% myeloblasts with increased eosinophils. Cytogenetic testing and immunotyping by fluorescent in situ hybridization (FISH) revealed inversion of chromosome 16, as well as 7q deletion in a small (5.7%) population of blasts. She was discharged with her cellulitis improved and then readmitted a week later to begin chemotherapy. On readmission, her CBC showed WBC 18.9 × 10^9^/L, Hgb 8.5 g/dL, and platelets 60 × 10^9^/L, with 4% circulating blasts noted. The patient was started on an induction regimen of cytarabine, daunorubicin, and etoposide per the COG AAML0531 protocol.

On day 4 from initiation of chemotherapy, our patient developed fever (Tmax 39.7°C), tachycardia (169/min), and tachypnea (100/min). Her WBC had dropped to 3.59 × 10^9^/L from 11.01 × 10^9^/L the day before. Serial chemistries, creatinine, and uric acid all remained within normal limits throughout. A chest X-ray at that time showed a patchy right middle lobe infiltrate concerning for pneumonia. Empiric antimicrobial coverage was instituted with IV ceftriaxone and vancomycin. Over the subsequent 24 hrs, the patient's respiratory status progressively deteriorated requiring transfer to the Pediatric Intensive Care Unit (PICU) for escalation of respiratory support. Chemotherapy was stopped at this time and antibiotic coverage was expanded with the addition of meropenem and caspofungin. Initially the patient was placed on high flow nasal cannula at 10 liters per minute with inhaled nitric oxide at 20 parts per million but quickly required intubation for ongoing oxygenation failure and respiratory acidosis. With positive pressure ventilation, the patient developed hemodynamic compromise with significant metabolic and lactic acidoses requiring dopamine and epinephrine continuous infusions. Due to ongoing hypoxia and hypercarbia, the patient was transitioned to high frequency oscillatory ventilation (HFOV) within 5 hours of initial intubation. Within one hour after placement on HFOV, the patient suffered a prolonged cardiopulmonary arrest (intermittent but persistent for approximately 2 hours) requiring compressions and multiple doses of epinephrine, calcium chloride, and sodium bicarbonate. The patient was ultimately placed on venoarterial ECMO by the general surgical team due to ongoing cardiopulmonary failure. Arterial blood gas measurement just prior to arrest showed pH 6.91, pCO_2_ 76, pO_2_ 53, HCO_3_ 15, base excess −21, and O_2_ sat 62%. This yielded a paO_2_ : FiO_2_ ratio of 53 and an oxygenation index of 49, both consistent with acute respiratory distress syndrome (ARDS). Chest radiograph obtained showed bilateral diffuse pulmonary opacification. Following the episode of cardiopulmonary arrest, the patient went into nonoliguric renal failure with doubling of creatinine (0.8 mg/dL) and development of hyperphosphatemia (12.2 mg/dL) and was placed on continuous venovenous hemodialysis (CVVHD) via the ECMO circuit.

Our patient had completed 6 of the planned 10 days of chemotherapy and the decision was made to defer the remainder of her first course of induction due to multiorgan failure. She had RSV upper respiratory infection on presentation at initial diagnosis 3 weeks before. A repeat respiratory viral panel PCR at the time of admission for initiation of induction chemotherapy was still positive for RSV. PCR from bronchoalveolar lavage (BAL) fluid at the time of respiratory compromise, however, tested negative for RSV. The remainder of her infectious workup failed to show an etiologic organism. The patient had a continuous electroencephalogram during days 1 through 4 of ECMO that demonstrated potential hypoxic-ischemic injury. Her echocardiogram (ECHO) performed while the patient was on ECMO showed elevated right-sided pressures (half systemic) but with normal structure, function, and systolic ejection fraction (LVEF 48.3%), similar to an ECHO obtained 24 hours prior to her arrest. She was weaned off both ECMO and CVVHD by day 9 and extubated by day 13. Hypersensitivity testing for both cytarabine and etoposide yielded negative results. Her leukemia was in remission by morphology, flow, and FISH at the end of induction I. She did not suffer any substantial or permanent neurologic sequelae from the cardiopulmonary arrest. Her second course of induction was given in the ICU with no resulting adverse events. She went on to receive the remainder of her chemotherapy cycles and remains in leukemia remission.

## 3. Discussion

Cytokine-release syndrome as a result of leukemia cell lysis has been previously elucidated as a possible mechanism behind early pulmonary complications seen in newly diagnosed AML patients [1–3]. It is speculated that cytokines known to be secreted by myeloblasts are released after cell lysis, either spontaneously or after initiation of chemotherapy, and exert their effect at the level of the lung endothelial cell [4]. Many myeloblast cell lines constitutively express proinflammatory cytokines not found in normal hematopoietic cells such as IL-1 beta, IL-6, IL-8, and TNF-alpha [5–7]. IL-1 beta and TNF-alpha have been identified in bronchoalveolar lavage fluids from patients with acute respiratory distress syndrome (ARDS) [8], an acute inflammatory process in the airspaces, and lung parenchyma that is not dissimilar to the lysis pneumopathy described in AML patients.

Similar to previous reports, the clinical picture in our patient was consistent with SIRS (fever, tachycardia, and respiratory distress) and occurred during rapid tumor reduction [3]. There were 2 notable differences, however, namely, (1) the severity of pulmonary compromise and (2) leukemia remission achieved in our patient despite cessation of treatment midinduction. Although our patient was positive for RSV on viral PCR testing by nasal swab, BAL testing was negative for RSV. As such, the positive result was felt to be due to ongoing viral shedding, which is not uncommon in patients undergoing chemotherapy. None of the previously described cases were severe enough to result in cardiac arrest and need for ECMO bypass. The presence of elevated right-sided pressures with an otherwise normal ECHO 24 hours prior to her arrest and while on ECMO led us to conclude that our patient's cardiac compromise was secondary to primary respiratory failure rather than from direct cardiotoxicity. While the patient did develop metabolic and lactic acidoses in the hours leading up to her arrest, this is most likely a result of anaerobic metabolism at the end-organ level due to profound hypoxemia associated with ARDS as evidenced by her paO_2_ : FiO_2_ ratio and oxygenation index just prior to her arrest. Furthermore, the introduction of positive pressure ventilation followed by HFOV was a contributing factor. By converting the intrathoracic cavity from a negative to a positive pressure chamber, more than likely the patient's overall hemodynamic status was acutely stressed with a drop in right-sided venous return to the heart as well as a potential abrupt increase in her pulmonary vascular resistance. This is in addition to the pulmonary hypertension she was exhibiting with right ventricular pressures half of her systemic systolic pressure. Though cytarabine has been reported to cause anaphylaxis [9] and proinflammatory cytokine release at higher doses [10], our patient had a negative skin test anergy and went on to receive subsequent cytarabine doses without further complications.

As this cell lysis-related phenomenon can occur during early chemotherapy for AML, particularly in M4/M4eo/M5 patients [3], vigilance with close monitoring is needed once induction treatment is started. Steroids with their added ability to induce leukemia cell apoptosis may be beneficial but their role needs to be clearly defined. Future studies looking at the cytokine expression profile during this phenomenon may be beneficial in identifying other candidate anti-inflammatory agents (e.g., tocilizumab, etanercept).

Unlike previously reported cases, our patient's symptoms did not improve quickly enough to allow resumption of induction therapy. Despite this, our patient went on to achieve complete hematologic and cytogenetic remission at the end of induction I. One possible explanation would be an antileukemia effect secondary to cytokine proliferation. In fact, several studies have demonstrated the potential role of cytokines in the treatment of AML [11]. The proinflammatory molecule IL-2, which enhances tumor cytotoxicity by inducing proliferation and activation of T and NK cells, is the cytokine that has been most extensively studied in adult as well as childhood AML [12, 13]. Though a rare phenomenon, various case reports have reported spontaneous remission of AML following episodes of infection [14–17], suggesting that triggered T cell-driven immune responses likely via cytokine activation can induce myeloid leukemia cell remission. More specifically, cytokines such as TNF and IL-2, both of which are released during active infections, have been suggested to play a potential role in inducing spontaneous AML remission [18, 19].
